# Development and validation of a web-based headache diagnosis questionnaire

**DOI:** 10.1038/s41598-022-11008-y

**Published:** 2022-04-29

**Authors:** Kyung Min Kim, A Ra Kim, Wonwoo Lee, Bo Hyun Jang, Kyoung Heo, Min Kyung Chu

**Affiliations:** 1 Department of Neurology, Severance Hospital, Yonsei University College of Medicine, 50-1 Yonsei-ro, Seodaemun-gu, Seoul, 03722 Republic of Korea; 2 Hankook Research, Seoul, Korea

**Keywords:** Neurology, Signs and symptoms

## Abstract

Information technology advances may help in conducting epidemiological studies using web-based surveys. Questionnaire-based headache diagnosis should be validated against the doctor’s diagnosis. This study aimed to develop and validate a web-based diagnostic questionnaire for migraine, probable migraine (PM), and tension-type headache (TTH). We constructed a seven-item questionnaire for diagnosing migraine, PM, and TTH. A web-based survey was conducted among adults aged 20–59 years; migraine, PM, and TTH were diagnosed based on the responses. Validation interview was performed via telephone by a neurologist within 1 month after the web-based interview. Finally, 256 participants completed both web-based survey and validation interview. Of them, 121 (47.3%), 65 (25.4%), 61 (23.8%), and 9 (3.5%) were diagnosed with migraine, PM, TTH, and unclassified headache (UH), respectively in the web-based survey, whereas 119 (46.5%), 60 (23.4%), 74 (28.9%), 2 (0.8%), and 1 (0.4%) were diagnosed with migraine, PM, TTH, UH, and primary stabbing headache, respectively in the validation interview. The best agreement was found in migraine (sensitivity: 92.6%; specificity: 94.8%; kappa coefficient: 0.875), followed by TTH (sensitivity: 78.4%; specificity: 98.4%; kappa coefficient: 0.809). PM showed the least agreement (sensitivity: 85.0%; specificity: 92.9%; kappa coefficient: 0.757). In conclusion, our questionnaire is valid in identifying these headache disorders.

## Introduction

The gold standard for diagnosing a headache disorder is via clinical interview by a neurologist using diagnostic criteria^1^. Diagnosis of headache using a self-administered questionnaire is an attractive method for clinical and epidemiological studies as it is inexpensive to conduct and relatively easy to use. Nevertheless, it should be evaluated for its validity by comparing it with the doctor’s diagnosis via clinical interview^2^. Several studies have been conducted for validating the diagnostic questionnaires for headache disorders^2–7^.

With recent advances in information technology, web-based surveys have been introduced and are increasingly used for a variety of purposes. Recently, web-based surveys have been used in epidemiological studies for diagnosing migraine and other headache disorders^8–10^. They showed similar findings to traditional interview surveys, but differences were also observed^10,11^. Chronic Migraine Epidemiology and Outcomes (CaMEO) was a web-based survey for migraine while the American Migraine Prevalence and Prevention (AMPP) study collected mailed questionnaires. Both studies sampled from panels representative of the population of the USA. The proportion of individuals who had chronic migraine was similar between the two studies (AMPP, 6.6%; CaMEO, 8.8%) while the response rate was higher in the AMPP (65.8%) study than in the CaMEO (16.5%) study.

The aims of the present study are: (1) To develop a web-based headache diagnostic questionnaire for common primary headache disorders including migraine, probable migraine (PM), and tension-type headache (TTH); (2) to validate a web-based headache diagnostic questionnaire by comparing it with the doctor’s diagnosis via telephonic interview.

## Results

### Participants

**Figure 1:**
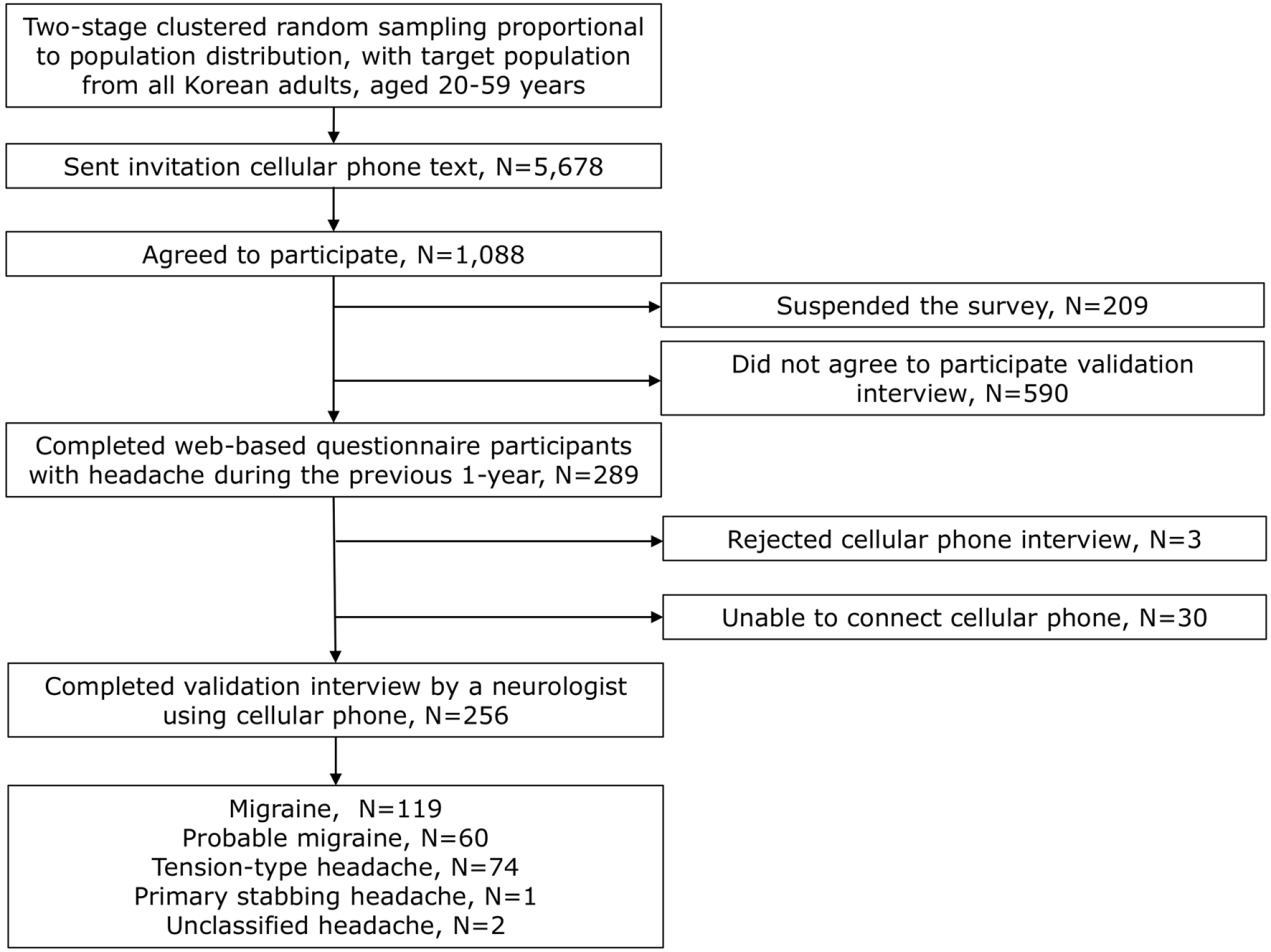
Flow chart depicting the participation of the individuals in this study.

The flow of participants is summarized in Fig. 1. Invitations for participation were sent via a mobile phone text to 5678 participants, and 1088 agreed to participate. Among them, 590 did not agree to participate in the validation interview, 209 rejected the web-based survey, and 289 completed the web-based survey. During the validation telephonic interview, 3 of them rejected the call, and 30 were unable to connect the call. Finally, 256 participants finished the validation interview. Of 256 participants, 135 (52.7%), 65 (25.4%), 61 (23.8%), and 9 (3.5%) were diagnosed with migraine, PM, TTH, and unclassified headache (UH), respectively in the web-based survey. Among the 65 participants with PM in the questionnaire-based diagnosis, 37 (56.9%) did not meet the criterion of typical headache characteristics, 19 (29.2%) did not meet the criterion of typical headache duration, and 9 (13.8%) did not meet the criterion of accompanying symptoms. In the validation interview, 119 (46.5%), 60 (23.4%), 74 (2.9%), 2 (0.8%), and 1 (0.4%) were diagnosed with migraine, PM, TTH, UH, and primary stabbing headache, respectively. Among 60 participants with PM in doctor’s diagnosis via telephone interview, 33 (55.0%) did not meet the criterion of typical headache characteristics, 19 (31.7%) did not meet the criterion of typical headache duration, and 8 (13.3%) did not meet the criterion of accompanying symptoms. One participant had a primary stabbing headache. Demographic and clinical characteristics of the participants are summarized in Table 1. The age and sex distribution of the participants were not significantly different from those in the general population of Korea (Supplementary Table S1).

**Table 1:** Demographics and clinical characteristics of the participants.

	Total N = 289	Completed interview N = 256	Migraine N = 119	Probable migraine N = 60	Tension-type headache N = 74
Age, mean (95% CI)	41.0 (31.5–51.0)*	41.0 (31.0–51.0)^†^	42.0 (32.0–50.0)^†^	37.0 (28.0–51.75)^†^	41.0 (33.0–51.25)^†^
Sex (women), N (%)	144 (49.8)*	128 (50.0)^†^	70 (58.8)^†^	30 (50.0)^†^	28 (37.8)^†^
**Headache intensity**					
Mild, N (%)	98 (33.9)*	55 (21.5)^†^	6 (5.0)^†^	11 (18.3)^†^	36 (48.6)^†^
Moderate, N (%)	169 (58.5)*	179 (69.9)^†^	96 (80.7)^†^	45 (75.0)^†^	37 (50.0)^†^
Severe, N (%)	22 (7.6)*	3 (8.6)^†^	17 (41.3)^†^	4 (6.7)^†^	2 (2.7)^†^
Headache frequency per month, median (range)	1.00 (0.33–2.00)*	2.00 (1.00–4.00)^†^	2.00 (1.00–3.00)^†^	2.00 (1.25–4.00)^†^	2.0 (1.00–4.00)^†^
Unilateral pain, N (%)	144 (49.8)*	147 (57.4)^†^	74 (62.2)^†^	32 (53.3)^†^	32 (52.7)^†^
Pulsating quality, N (%)	129 (44.6)*	170 (66.4)^†^	93 (78.2)^†^	42 (70.0)^†^	35 (47.3)^†^
Aggravation by routine physical activity, N (%)	137 (47.4)*	131 (51.2)^†^	85 (71.4)^†^	33 (55.0)^†^	13 (17.6)^†^
Nausea, N (%)	186 (64.4)*	123 (48.0.0)^†^	95 (79.8)^†^	28 (46.7)^†^	0 (0.0)^†^
Vomiting, N (%)	47 (16.3)*	21 (8.2)^†^	17 (14.3)^†^	4 (6.7)^†^	0 (0.0)^†^
Photophobia, N (%)	113 (39.1)*	76 (29.7)^†^	58 (48.7)^†^	17 (28.3)^†^	1 (1.4)^†^
Phonophobia, N (%)	189 (65.4)*	109 (42.6)^†^	70 (58.8)^†^	28 (46.7)^†^	11 (14.9)^†^

*N* number, *CI* confidence interval.

*Results from the web-based survey.

^†^Results from the telephonic validation interview.

### Validity of headache diagnoses

The sensitivity, specificity, and positive and negative predictive values of migraine, PM, and TTH diagnosis and agreement with kappa statistics between the web-based survey and validation interview of all 256 participants are shown in Table 2. Kappa coefficients for migraine and TTH showed very good agreements whereas that for PM showed a good agreement (Table 2).

**Table 2:** Diagnostic validity of migraine, probable migraine, and tension-type headache of all 256 participants.

	Sensitivity	Specificity	Positive predictive value	Negative predictive value	Accuracy	Kappa coefficient
Migraine, % (95% CI)	92.6 (86.4–96.5)	94.8 (89.6–97.9)	94.1 (88.6–97.1)	93.4 (88.3–96.4)	93.8 (90.1–96.4)	0.875 (0.816–0.934)
Probable migraine, % (95% CI)	85.0 (73.4–92.9)	92.9 (88.3–96.0)	78.5 (68.5–85.9)	95.3 (91.7–97.4)	91.0 (86.8–94.2)	0.757 (0.663–0.870)
Tension-type headache, % (95% CI)	78.4 (67.3–87.1)	98.4 (95.3–99.7)	95.1 (86.2–98.4)	91.8 (87.9–94.5)	92.6 (88.7–95.7)	0.809 (0.727–0.851)

*CI* confidence interval.

We also conducted analyses among 101 participants with low frequency headaches (< 1 attack/month) (Table 3). Kappa coefficient for migraine revealed a very good agreement and those for PM and TTH demonstrated a good agreement. A subgroup analysis among 32 participants with high frequency headaches (> 6 attacks/month) showed a very good agreement for migraine and good agreements for PM and TTH (Table 4).

**Table 3:** Diagnostic validity of migraine, probable migraine, and tension-type headache of 101 participants with low frequency (< 1 attack/month) headache.

	Sensitivity	Specificity	Positive predictive value	Negative predictive value	Accuracy	Kappa coefficient
Migraine, % (95% CI)	92.9 (66.1–99.8)	88.9 (65.3–98.6)	86.7 (63.6–96.0)	94.1 (70.6–99.1)	90.6 (75.0–98.0)	0.811 (0.607–1.000)
Probable migraine, % (95% CI)	81.8 (48.2–97.7)	95.2 (76.2–99.9)	90.0 (56.6–98.4)	90.9 (74.0–97.2)	90.6 (75.0–98.0)	0.788 (0.560–1.000)
Tension-type headache, % (95% CI)	100.0 (59.0–100.0)	100.0 (86.3–100.0)	100.0 (100.0–100.0)	100.0 (100.0–100.0)	100.0 (89.1–100.0)	1.000 (1.000–1.000)

*CI* confidence interval.

**Table 4:** Diagnostic validity of migraine, probable migraine, and tension-type headache of 32 participants with high frequency (> 6 attacks/month) headache.

	Sensitivity	Specificity	Positive predictive value	Negative predictive value	Accuracy	Kappa coefficient
Migraine, % (95% CI)	92.9 (66.1–99.8)	88.9 (65.3–98.6)	86.7 (63.6–96.0)	94.1 (70.6–99.1)	90.6 (75.0–98.0)	0.811 (0.607–1.000)
Probable migraine, % (95% CI)	81.8 (48.2–97.7)	95.2 (76.2–99.9)	90.0 (56.6–98.4)	90.9 (74.0–97.2)	90.6 (75.0–98.0)	0.788 (0.560–1.000)
Tension-type headache, % (95% CI)	100.0 (59.0–100.0)	100.0 (86.3–100.0)	100.0 (100.0–100.0)	100.0 (100.0–100.0)	100.0 (89.1–100.0)	1.000 (1.000–1.000)

*CI* confidence interval.

## Discussion

We developed a seven-item web-based diagnostic questionnaire for migraine, PM, and TTH based on the ICHD-3 criteria^12^. We found that our questionnaire showed very good or good agreement in the diagnosis of migraine, PM, and TTH when compared to a neurologist’s diagnoses.

Various studies have been conducted for validating a questionnaire in the diagnosis of migraine^2–7^ by comparing questionnaire-based diagnoses and clinical interview-based diagnoses. Most studies have shown that the kappa coefficients range from 0.43 to 0.77, which suggests a moderate to good agreement. However, our study showed a kappa coefficient of 0.875, which suggests a very good agreement. The high rate of agreement in diagnosing migraine was also confirmed in the subgroup analyses. Therefore, we could confirm that we had developed a highly valid questionnaire for diagnosing migraines.

Web-based surveys have several aspects that differ from those of the traditional interview surveys. They need shorter time, less cost to conduct, and make the access to unique populations and data quality control easy. Moreover, real-time access is available and interviewers are not required for these surveys^13^. Its disadvantages include survey fraud, limited sampling and respondent availability, difficulty in generating samples from an online community, and less experience of the individuals in undergoing a web-based survey^14^. For the optimal use of the web-based survey questionnaire, its validity should be properly evaluated^2^. Nevertheless, the validation of a web-based migraine or headache diagnostic questionnaire has rarely been reported. An American study, Penn Online Evaluation of Migraine, evaluated a web-based questionnaire for diagnosing migraines, which included 59 individuals with migraine and 31 headache-free control, and found a sensitivity of 59% and a specificity of 84% in diagnosing migraines^15^. The present study included sufficient sample size and showed that our questionnaire had a very good or good agreement in the diagnosis of migraine, PM and TTH.

Our study found that the diagnostic accuracy of PM was lower compared to that of migraine an TTH. Similar results were also found in a computer-based questionnaire study in China^16^. The study revealed that Kappa coefficients of PM and probable TTH were 0.698 and 0.639, respectively when compared to doctor’s diagnosis. In contrast, Kappa coefficients of migraine and TTH were 0.961 and 0.921, respectively. Therefore, it should be noted that the diagnostic accuracy of ‘probable’ headache diagnosis was lower from that of ‘strict’ headache diagnosis when questionnaire-based headache diagnosis applied.

A better agreement was noted in cases where there was a shorter time interval between the initial survey and the validation interview^7^. A guideline for headache epidemiology study recommended that re-interviewing should not be conducted more than once in a month^1^. All validation interviews were performed within 1 month after the web-based survey in the present study. This short interval between the initial survey and validation interview was made possible by the web-based survey, and it might have contributed to the high agreement observed in this study.

Web-based headache diagnosis could be useful in several ways in addition to clinical and epidemiological studies. Approximately half of individuals with migraine did not know their migraine diagnosis^17,18^. Web-based diagnostic questionnaire can be a way to increase awareness of migraine diagnosis. Clinical decision systems have shown effectiveness for improving clinical practice^19,20^. Thus, a clinical decision system based on the headache diagnosis using web-based questionnaire would be a good choice in the management of headache^16,21,22^. Also, web-based questionnaire could promptly assess changes in headache characteristics and in treatment strategies during public health emergency situation like coronavirus disease 2019 pandemic^23,24^. Our web-based questionnaire showed a high diagnostic accuracy for migraine, PM and TTH, and will provide a good opportunity not only in the diagnosis of headache but also in the management of headache disorders.

The present study has several limitations. First, the distribution of headache diagnosis was different from that of the general population. Migraine prevalence in the present study was 46.4%, which was higher than that in epidemiological studies in Asian countries, which ranges between 5.1 and 9.0%. A higher prevalence of migraine in this study might be attributed to the recruitment bias in the web-based survey^1,25^. Individuals with migraine might have more interest in a headache survey than those without migraine and this could have resulted in a higher prevalence of migraine in the present study. Although some validation studies of headache diagnostic questionnaires were conducted in populations with different headache characteristics from the main survey population^2,7^, the validity should be evaluated in the relevant populations for the proper use of the web-based survey questionnaires. Second, we did not assess the demographic features of non-participating individuals. Identifying characteristics of non-participating individuals is an important issue in population-based studies^26^. Nevertheless, we could not obtain demographic characteristics of non-participating individuals owing to the personal information protection act in Korea. Third, we included a sufficient sample size of participants for the evaluation of the diagnostic validity of migraine, PM, and TTH in the present study. Nevertheless, optimal sample size for some subgroup analyses might not have been maintained. Although we found that our questionnaire showed good or very good agreements in the participants with low frequency and high frequency headaches, this point is a limitation of our study. Lastly, the overall cooperating rate in the present study was not high. We recruited participants who had at least one headache attack during the previous 1 year and agreed to participate in the validation interview via a mobile phone. These conditions might have contributed to the low cooperation rate. The lower cooperation rate in a web-based survey than that in a traditional mail survey has already been reported^8,11^. Nevertheless, the age and sex distribution of our participants were not significantly different from that in the total population of Korea.

The present study also has several strengths. First, we developed and validated a diagnostic questionnaire for PM in combination with migraine and TTH. PM is a common headache disorder, and its symptoms and disabilities are similar or somewhat milder than those of migraine^26^. Some epidemiological study evaluated its prevalence and clinical characteristics using a questionnaire. Nevertheless, diagnostic validity of the questionnaire has not been specifically evaluated yet. This is the first validation study for the diagnosis of PM. Second, we included enough participants to evaluate the sensitivity and specificity of the web-based questionnaire in diagnosing migraine, PM, and TTH. By having sufficient sample size in the present study, we can confirm that the diagnostic validity of our web-based questionnaire has been properly evaluated.

In conclusion, we developed a web-based questionnaire for diagnosing migraine, PM, and TTH based on the ICHD-3 criteria. By comparing the web-based survey diagnosis and the diagnosis by a neurologist, we found that our web-based questionnaire showed a very good agreement for migraine and TTH and a good agreement for PM. The findings of this study suggest that our web-based headache diagnostic questionnaire is a valid tool for diagnosing migraine, PM, and TTH.

## Methods

### Development of headache diagnosis module

We developed a questionnaire for the headache diagnosis based on the ICHD-3 criteria of migraine, PM, and TTH. Since both criteria of migraine without (code 1.1) and with aura (code 2.1) were composed of five criteria including the total number of attacks, duration of attacks, typical headache characteristics, accompanying symptoms, and a poor accountability by another headache diagnosis questionnaire, we constructed seven questions for the duration of attack, typical headache characteristics, and accompanying symptoms^12^. We included a question for headache attacks about typical attack duration. For typical headache characteristics and accompanying symptoms, we incorporated five questions regarding headache intensity, unilateral pain, pulsating quality, aggravation by routine physical activity, nausea, vomiting, photophobia, and phonophobia (Table 5 and Supplementary Table S2). A question for headache frequency was also included.

**Table 5:** Web-based questionnaire for the diagnosis of headache.

These questions about your “most bothersome headache” during the previous 1 year			
Q1. On an average, how long did these headaches last?			
	(——) second (s), (——) minute (s), (——) hour (s), (——) day (s),		
Q2. How often did you experience such headaches during the last 1 year?			
	(1) (——) attacks in 1 year (2) (——) attacks in 1 month		
	(3) (——) attacks in 1 week (4) (——) attacks in 1 day (5) (——) attacks in 1 h		
Q3. How bad was your headache?			
	(1) Headache did not disturb usual daily activities (mild)		
	(2) Headache often disturbed usual daily activities, but I could perform more than half of my daily activities (moderate)		
	(3) I can’t perform my usual daily activities when I suffer these headaches (severe)		
Q4. What was the location of the headache?			
	(1) Right side	(2) Left side	
	(3) Bilateral sides	(4) whole head	
	(5) Unilateral either way		
Q5. What was the headache like? Please statement all describes your headache during the previous year			
	(1) Pulsating and throbbing	(2) Exploding	
	(3) Pounding or like a pulse		
	(4) Tightening feeling like tying a band around your head		
	(5) Sharp like pinpricking	(6) Sudden and severe like hitting your head with a hammer	
	(7) Other: describe		
Q6. These are questions asking about your headaches. (No. 1–5)			
Question No		Yes	No
1	Do you feel sick to your stomach during your headaches?		
2	Do you feel nauseated during your headaches?		
3	Do you vomit during your headaches?		
4	Does light bother you a lot more than when you didn’t have headaches?		
5	Is your headache more painful when you are in noisy surroundings?		
Q7. The headache is worsened by activities such as walking or climbing stairs?			
	(1) Yes		
	(2) No		

We did not consider criteria A and E for the total number of attacks and a poor accountability by another headache diagnosis questionnaire in migraine without aura and infrequent episodic TTH. Most individuals with headaches could not recall the exact number of headache attacks. Moreover, adult individuals with at least one attack of headache during the previous 1 year may have had experienced more than 10 headache attacks during their lifetime. Approximately 98% of individuals with headaches had primary headache disorders, and it is very difficult to diagnose headaches other than common primary or secondary headache disorders in epidemiological studies^12,27^.

### Participants

We recruited participants for the present study via a mobile phone text invitation among the survey panels of Hankook Research. An invitation mobile phone text was sent with the title ‘a survey on headaches’. The invitation text messaging was not stratified by sex or age. The inclusion criteria were: (1) aged 20–59 years (2) having at least one headache attack during the previous 1 year, (3) able to participate in the web-based headache survey, and (4) agreed to participate in an additional telephonic interview by a doctor. Exclusion criteria were as follows: (1) aged < 20 or > 59, (2) did not experience any headache attack during the previous 1 year, and (3) did not agree to participate in an additional telephonic interview.

### Web-based survey

After accepting the participation request, internet link for the web-based survey was sent to all the participants, and they were asked to complete the survey. The web-based survey was conducted from May 1 to May 10, 2021.

### Validation interview

A semi-structured telephonic validation interview was performed by a neurologist with a special interest in headache medicine (MKC) using a case report form (Supplementary Table S2). All participants were asked about (1) the occurrence of headache during the previous 1 year, (2) duration of headache attacks, (3) intensity of headache, (4) typical headache characteristics including unilateral pain, pulsating quality, aggravation by routine physical activity, and (5) accompanying symptoms of nausea, vomiting, photophobia, and phonophobia. If the telephonic interview call was not connected, the interviewer tried to call up to three times. Validation interview was performed between May 14 to May 31, 2021.

### Sample size calculation

Sample size calculation was based on the prevalence, confidence interval, and the estimated sensitivity and specificity of the questionnaire for diagnosing migraine, PM, and TTH. We set the estimated prevalence of migraine, PM, and TTH as 15%, 20%, and 60%, respectively among participants’ headaches based on the previous epidemiological studies^28–30^. To achieve a maximal error of 10%, the sample size for 90% sensitivity and 95% specificity for migraine, PM, and TTH was calculated as 230, 172, and 57, respectively^31^. Therefore, we set the target sample size as 230 in the present study.

### Identification of the duration, intensity, characteristics, and accompanying symptoms of headache

We identified the duration, intensity, characteristics, and accompanying symptoms of headache based on the responses to the questionnaire (Table 5 and Supplementary Table S2). The duration and intensity of headache were determined based on the response to Q1 and Q3, respectively. Unilateral pain was considered by positive responses to (1), (2), or (5) of Q4. Pulsating quality was recognized by positive responses to (1) or (3) of Q5. Aggravation by routine physical activity was identified by a positive response to Q7. Nausea, vomiting, photophobia, and phonophobia were assigned when a positive response was recorded to (2), (3), (4), and (5) questions of Q6, respectively.

### Diagnoses of migraine, PM, and TTH

Diagnoses of migraine, PM, and TTH in the web-based survey were performed based on the ICHD-3 criteria. If a participant’s headache fulfilled B-D criteria of migraine without aura (code 1.1), the participant was classified as having migraine. The participant who met all but one criterion of the migraine diagnostic criteria was classified as having PM (code 1.5). Since it is very difficult to accurately identify the presence of aura using questionnaire, we did not evaluate the presence of aura^32^. Therefore, participants with migraine in the present study included both migraine with and without aura. Similarly, PM included both PM with (code 1.5.2) and without (code 1.5.1) aura.

If a participant’s headache fulfilled all but one criteria of migraine, the participant was diagnosed as having PM. Diagnoses of TTH was based on criteria B through D for infrequent TTH (code 2.1) in ICHD-3. Participants who met all these criteria were considered to have TTH. We did not apply the frequency criterion (criterion A) in the diagnosis of TTH. Thus, the TTH evaluated in this study included infrequent TTH (code 2.1), frequent TTH (code 2.2), and chronic TTH (code 2.3). When a participant was diagnosed with TTH and PM simultaneously, a diagnosis of TTH was assigned^12^. If a participant’s headache did not fulfill the criteria for migraine, PM, and TTH, the participant was diagnosed with an UH.

During the validation interview, all headache diagnostic criteria for migraine (code 1.1, criteria A–E), PM (code 1.5, criteria A–C), and TTH (code 2.1, criteria A–E) were applied. Headache diagnoses other than migraine, PM, TTH, and UH were also allowed.

### Ethical considerations

This study was approved by the institutional review board/ethics committee of Severance Hospital, Yonsei University (IRB No. 2021-0538-001). Written informed consent was obtained from each participant. All procedures involving human participants were performed in accordance with the ethical standards of the institutional and/or national research committee as well as the tenets of the 1964 Declaration of Helsinki and its later amendments, or comparable ethical standards.

### Statistical analyses

Sensitivity, specificity, positive and negative predictive values, accuracy, and kappa coefficients with 95% confidence interval were calculated for different headache diagnoses based on the responses in the web-based questionnaire using a neurologist’s headache diagnoses as a gold standard. A kappa coefficient of ≤ 0.20 was considered as poor, between 0.21 and 0.40 as fair, between 0.41 and 0.60 as moderate, between 0.61 and 0.80 as good, and between 0.81 and 1.00 as a very good agreement^33^.

## Supplementary Information

Supplementary Information.

## Data Availability

Anonymized data relevant to this study will be shared by request with qualified investigator pending appropriate Institutional Review Board approvals.
