# Supplementary material for: Development and validation of a web-based headache diagnosis questionnaire
Source: Sci Rep. 2022 Apr 29;12:7032. doi: 10.1038/s41598-022-11008-y (PMC9052186; doi:10.1038/s41598-022-11008-y)
Supplement: Supplementary file 1 — Supplementary Information. [file 41598_2022_11008_MOESM1_ESM.docx]

**Development and validation of a web-based headache diagnosis questionnaire**

Kyung Min Kim, MD^1^; A Ra Kim^2^; Wonwoo Lee, MD^1^; Bo Hyun Jang^2^; Kyoung Heo, MD, PhD^1^; Min Kyung Chu, MD, PhD^1*^

**Supplementary Table S1.** Age and sex distribution of web-based survey participants and the total population of Korea

|  | | Survey participants N (%) | Total population N (%) | *p* value |
| --- | --- | --- | --- | --- |
| Sex | |  |  | 0.865 |
|  | Men | 128 (50.0) | 15,529,105 (51.2) |  |
|  | Women | 128 (50.0) | 14,778,651 (48.8) |  |
| Age groups | |  |  | 0.818 |
|  | 20-29 years | 54 (21.1) | 6,719,119 (22.1) |  |
|  | 30-39 years | 65 (25.4) | 6,839,377 (22.6) |  |
|  | 40-49 years | 56 (21.9) | 8,208,901 (27.1) |  |
|  | 50-59 years | 81 (31.6) | 8,540,359 (28.2) |  |
| Total | | 256 | 30,307,756 (100.0) |  |

**Supplementary Table S2.** Case report form used in the semi-structured telephonic validation interview

**Case Report Form**

ID Mobile phone number Name

1. **Average duration of headache attacks:**

**On an average, how long did these headaches last?**

(<30 min.) (30 min.-4 h) (4 – 72 h) (≥72 hours)

1. **Frequency of headache attacks:**

**How often did you experience such headaches during the last 1 year?**  per month

1. **Intensity of headache: How bad was your headache?**

⬜ Mild: Headache did not disturb usual daily activities

⬜ Moderate: Headache often disturbed usual daily activities, but I could perform more than half of my daily activities

⬜ Severe: I can’t perform my usual daily activities when I suffer these headache

**Headache characteristics:**

**What was the location of the headache?**

⬜ Unilateral location ⬜ Bilateral location

**What was the headache like?**

⬜ Pulsating quality ⬜ Non-pulsating quality

**The headache is worsened by activities such as walking or climbing stairs?**

⬜ Aggravation by or causing avoidance of routine physical activity

⬜ Non-aggravation by or causing avoidance of routine physical activity

1. **Accompanying symptoms:**

**Do you feel nauseated during your headaches?** ⬜ Nausea

**Do you feel sick to your stomach during your headaches?** ⬜ Nausea

**Do you vomit during your headaches?**  ⬜ Vomiting

**Does light bother you a lot more than when you didn’t have headaches?** ⬜ Photophobia

**Is your headache more painful when you are in noisy surroundings?** ⬜ Phonophobia

**Results of the telephone interview diagnosis**

⬜ Migraine

⬜ Probable migraine

⬜ Not fulfilling typical duration

⬜ Not fulfilling typical characteristics

⬜ Not fulfilling accompanying symptoms

⬜ Tension-type headache

⬜ Other classified headaches

⬜ Unclassified headache
